# In search of a pan-coronavirus vaccine: next-generation vaccine design and immune mechanisms

**DOI:** 10.1038/s41423-023-01116-8

**Published:** 2023-12-26

**Authors:** S. Cankat, M. U. Demael, L. Swadling

**Affiliations:** Division of Infection and Immunity, Institute of Immunity and Transplantation, University College London, Pears Building, London, NW3 2PP UK

**Keywords:** Vaccine, Coronavirus, Cross-reactive immunity, Cross-protection, Universal, Vaccines, Viral infection, Cellular immunity, Humoral immunity

## Abstract

Members of the *coronaviridae* family are endemic to human populations and have caused several epidemics and pandemics in recent history. In this review, we will discuss the feasibility of and progress toward the ultimate goal of creating a pan-coronavirus vaccine that can protect against infection and disease by all members of the coronavirus family. We will detail the unmet clinical need associated with the continued transmission of SARS-CoV-2, MERS-CoV and the four seasonal coronaviruses (HCoV-OC43, NL63, HKU1 and 229E) in humans and the potential for future zoonotic coronaviruses. We will highlight how first-generation SARS-CoV-2 vaccines and natural history studies have greatly increased our understanding of effective antiviral immunity to coronaviruses and have informed next-generation vaccine design. We will then consider the ideal properties of a pan-coronavirus vaccine and propose a blueprint for the type of immunity that may offer cross-protection. Finally, we will describe a subset of the diverse technologies and novel approaches being pursued with the goal of developing broadly or universally protective vaccines for coronaviruses.

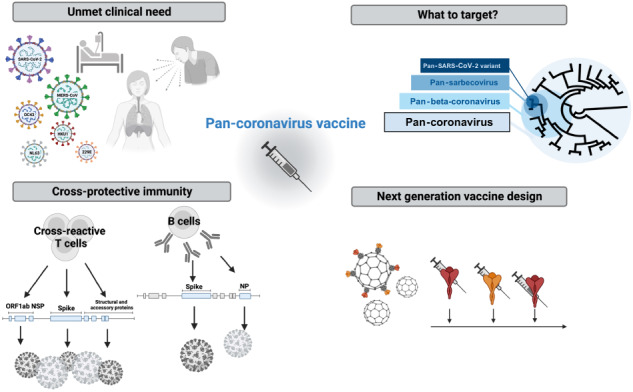

## Introduction

The COVID-19 pandemic has highlighted the significant effect that *emerging viral infections can have on global health*. Simultaneously, the pandemic has established the *positive impact that effective vaccine strategies* can have on mitigating the global burden of disease and highlighted the importance of understanding the *immune mechanisms underpinning protective vaccines*.

In the three and a half years since the identification of severe acute respiratory syndrome coronavirus 2 (SARS-CoV-2), ~770 million (M) infections and ~7 M related deaths have been reported [1]; furthermore, these data are likely gross underestimates of the true global impact of the virus. The panel of 18 World Health Organization (WHO)-licensed vaccines changed the course of the pandemic, and these vaccines are estimated to have prevented between 14 and 20 M deaths in their first year alone [2]. Despite the WHO declaring that COVID-19 was no longer a global emergency as of May 2023, there is still a significant unmet clinical need and global economic cost associated with currently circulating variants of SARS-CoV-2. Accordingly, there is a strong consensus in the scientific community regarding the need to develop more effective next-generation vaccines [3, 4].

The COVID-19 pandemic has also renewed interest in developing pan-family or universal vaccines—i.e., vaccines that could offer broad protection against all members of a viral family. The key rationale for a universal vaccine approach is the continued burden of disease associated with endemic human coronaviruses (HCoVs), the unpredictability of future SARS-CoV-2 variants, and the potential for zoonotic spill-over events. Since three of the major pandemics in the past two decades have been caused by coronaviruses, the demand for a proactive approach to coronavirus vaccines is especially warranted.

In this review, we will first outline the *need for a pan-coronavirus vaccine* and then focus on knowledge derived from *cross-reactive and heterosubtypic immunity* in SARS-CoV-2 infection. We will discuss how this knowledge has shed more light on the feasibility of developing a pan-coronavirus vaccine. Finally, we will provide an update on current progress regarding the use of repurposed or novel approaches for *next-generation coronavirus vaccines*.

## What is a pan-coronavirus vaccine?

**Fig. 1:**
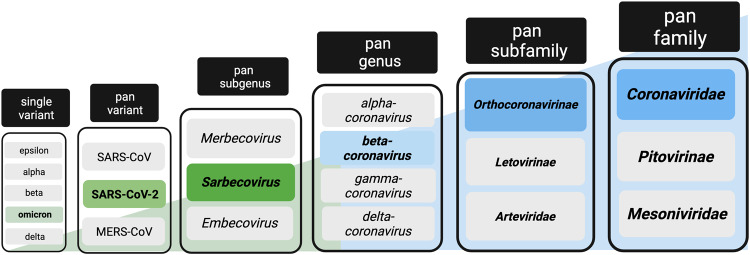
Phylogeny of coronaviruses and the breadth of protection afforded by different types of vaccines. Updated vaccines containing Omicron BA.1 or later sequences of spike have been designed to offer improved *single-variant* protection against currently circulating strains of SARS-CoV-2 compared with licensed vaccines. Next-generation vaccines are being designed to offer broader protection, for instance, *pan-variant* vaccines that could target all variants of the single viral species, SARS-CoV-2; *pan-subgenus* vaccines that target all members of the subgenus sarbecoviruses (including also SARS-CoV-1); *pan-genus* vaccines that include all members of the genus β-coronavirus (further including MERS-CoV, HCoV HKU1 and OC43); *pan-subfamily* vaccines that target all Orthocoronavirinae (also including alpha-coronaviruses HCoV NL63 and 229E); and *pan-coronavirus* vaccines that target all species within the Coronaviridae family. Created with BioRender.com

A pan-coronavirus vaccine is a vaccine that is effective at preventing severe disease and/or infection caused by all viruses of the coronavirus family (Fig. 1). In contrast, the current widely employed SARS-CoV-2 vaccines adopt a narrow-spectrum approach – they deliver spike (S) glycoproteins that vary antigenically between strains, resulting in immune protection that is predominantly species-specific or even variant-specific [5].

An intermediate goal has been to develop vaccines against a subset of these viruses, referred to as *broadly protective* coronavirus vaccines rather than *pan-* or *universal* vaccines. The *Coronaviridae* family of positive-stranded RNA viruses contains three subfamilies, including the *Orthocoronavirinae* subfamily, to which SARS-CoV-2 belongs. The *Orthocoronavirinae* subfamily is further divided into four genera (*α*, β, *γ*, δ; Fig. 1). Several pan-coronavirus approaches are in development, which will be discussed in detail below. These approaches include either targeting all *β*-coronaviruses, targeting a subset of these viruses (such as sarbecoviruses), or even targeting a single *species* with the aim of developing a pan-variant or “variant-proof” vaccine for SARS-CoV-2 (Table 1).

**Table 1:** Key characteristics of an ideal pan-coronavirus vaccine and the relative performance of current best-licensed vaccines

	Ideal pan-coronavirus vaccine	Current best in class licensed vaccine (example mRNA or Ad spike)
Breadth	All coronavirus family including all current HCoV, SARS-CoV-2 (all variants) and emerging animal coronaviruses	Largely strain/variant specific
Protection conferred	Protection from infection, transmission, and disease	Protection from severe disease, limited protection from infection
Durability	Sustained protection from a single dose	Requires boosters every 6–12 months
Safety Profile	Mild reactogenicity, no severe adverse events	Mild-moderate reactogenicity (mRNA>Ad), rare severe events (e.g., thrombosis with thrombocytopenia syndrome)
Patient Population	Universal (including immunocompromised)	Potentially universal, boosting now in clinically vunerable only
Route of Administration	Needle free, mucosal targeting?	Intradermal
Manufacturing	Scalable for global roll-out	Scalable for global roll-out
Storage	Room temperature	Adenovirus 2–8’C 1 year [46]
Cost per dose	Inexpensive and not-for-profit	Adenovirus $2–5, mRNA $10–25 [47]

### Why is there a clinical need for a pan-coronavirus vaccine?

There are six coronaviruses that continue to cause disease in humans: SARS-CoV-2, Middle Eastern respiratory syndrome coronavirus [MERS-CoV], and the four seasonal endemic HCoVs (OC43, HKU1, 229E, and NL63). Prophylactic vaccines would be beneficial in reducing the morbidity caused by these viruses. The clinical spectrum of disease caused by these virus ranges from asymptomatic infection to severe disease, characterized by pneumonia, dyspnea, and febrile illness, progressing in severe cases into acute respiratory distress, sepsis, and death from multiple organ failure [6]. The requirement for hospitalization and mechanical ventilation in patients with severe COVID-19 (mostly in high-risk groups, such as elderly individuals or those with comorbidities) caused acute strains on healthcare systems at the peak of the pandemic, thus leading to national lockdowns and vaccination programs.

The chronic morbidity burden of COVID-19 extends much wider, with up to 10% of individuals across a spectrum of initial disease severity estimated to suffer from long COVID, a durable multisystemic condition with multiple adverse outcomes, such as, myalgia, fatigue, and neurocognitive dysfunction [7]. *The underlying mechanisms and the immunopathology of long COVID-19 are a continued area of intense investigation* [8, 9].

Four seasonal human coronaviruses were circulating widely prior to the COVID-19 pandemic, accounting for ~10–30% of upper respiratory tract infections [10]. However, these seasonal coronaviruses typically cause self-limited infection and asymptomatic or mildly symptomatic disease. As a result, vaccine development was deemed to be a low priority, and it was thought that due to concurrent circulation, a quadrivalent vaccine would be needed. There is, however, renewed interest in HCoV-targeting vaccines. Moderna has developed an mRNA vaccine, mRNA-1287, to target the four seasonal HCoVs. Although no coronavirus vaccines had obtained regulatory approval prior to the SARS-CoV-2 pandemic, a vaccine protecting against seasonal coronaviruses could have enormous economic and health benefits globally [11].

MERS-CoV persists in dromedary camels in Middle Eastern countries, causing sporadic outbreaks in humans with limited human-to-human transmission. However, MERS-CoV has a case fatality rate of ~33% across 2500 cases [12]; thus, there is continued interest in a prophylactic vaccine [13].

Vaccines continue to be our best tool to limit the impact of SARS-CoV-2. For example, there remain >17.5 M at-risk individuals and their contacts (>65 yrs old, care home residents, clinical risk groups and their carers/household contacts, frontline health workers, and social care workers) who received booster vaccinations prior to the winter of 2022 in the UK, and a similar number of individuals were offered boosters in 2023 [14]. A pan-coronavirus vaccine would have wide-ranging impact by addressing an important unmet clinical need for endemic and epidemic coronaviruses.

### Could vaccines be designed to target emerging coronaviruses?

The urgent need for a pan-coronavirus vaccine, as opposed to a narrow-range SARS-CoV-2 vaccine, is underscored by the high probability of the continued emergence of coronaviruses with pathogenic potential [15].

The ability of viral variants to evade immunity was brought to the center stage by the emergence of the Omicron strain of SARS-CoV-2, which was first reported in November 2021; this variant harbored >30 amino acid substitutions within spike, thereby producing conformational changes that resulted in the evasion of recognition by neutralizing antibodies [16]. Subsequently, several reports confirmed that vaccines containing ancestral Wuhan-Hu-1 spike sequences only conferred limited protection against infection by Omicron [5, 17], which soon became one of the dominant global strains.

The emergence of viruses with novel immunogenic properties is neither surprising nor unexpected; rather, it reflects the *modus operandi* of viruses, which are sustained by cycles of diversification and adaptation.

At the molecular level, SARS-CoV-2, similar to other RNA viruses, uses an intrinsically imprecise RNA-dependent RNA polymerase, resulting in very high mutation rates [18]. At the ecological level, coronaviruses have broad host tropism across birds and mammals, with evidence of extensive transmission between species. Numerous bat species transmit sarbecoviruses to one another and to numerous mammals, including humans; among the nine coronaviruses known to infect humans, five are most likely to have originated from bats [15, 19]. Mixed infection within a single host opens the window for further diversification by genetic recombination.

Zoonotic events are probably highly underestimated due to poor surveillance [20]. For instance, MERS-CoV first caused an epidemic in humans after zoonotic transmission in 2012, although serological evidence suggests that it had been circulating in dromedary camels since the 1980s. Multiple separate cases of cattle coronaviruses being transmitted to humans have been described [21], and viruses related to feline and canine coronaviruses have been isolated from patients with respiratory symptoms [22, 23]. A porcine δ-coronavirus was isolated from three Haitian children with acute undifferentiated fever, which was the first occurrence of a δ-coronavirus in humans [24]. Surveillance of wild animals has also revealed high seropositivity rates against SARS-CoV-2 in white-tailed deer in the United States [25]. Anthroponosis (human to animal transmission) and reverse anthroponosis (spill-over back into humans after a period of adaptation in the new animal reservoir) have been detected for SARS-CoV-2, thus impeding the eradication of SARS-CoV-2 and increasing the risks of recombination, evolution and immune evasion [15].

We currently have little understanding of the universe of potentially endemic coronaviruses circulating in animal hosts, although a combination of high mutagenic properties and broad host range means that there is a persistent threat of novel zoonotic emergence. Furthermore, SARS-CoV-2 diversification is ongoing within human populations.

Due to the wide-ranging impact of the COVID-19 pandemic, there has been a renewed interest in *proactive vaccinology*, in which research efforts are concentrated on designing vaccines that can induce immunity that would cross-protect against future zoonosis. A pan-coronavirus vaccine could therefore represent the first in a line of “proactive vaccines” that could be ready for rapid testing during emerging outbreaks.

A pan-coronavirus vaccine approach could mitigate the substantial costs associated with repeated development and distribution of potentially ineffective vaccines in the face of an ever-changing viral landscape. A pan-coronavirus vaccine thus represents an *ultimate goal*. Below, we will discuss how the study of SARS-CoV-2 has reignited interest in a pan-coronavirus vaccine, what this vaccine might look like, and some of the approaches being pursued.

## What have we learned from first-generation vaccines?

### Against the emerging virus

Several complete SARS-CoV-2 viral genomes were available at the beginning of 2020 [26], and early analysis suggested very little sequence diversity [15]. This informed the rapid development of vaccines even before the pandemic potential of SARS-CoV-2 was predicted by many. The front-runners were vaccines that were based around these ancestral sequences and included only the surface glycoprotein spike.

With just a few exceptions, licensed vaccines for viruses are designed to *induce neutralizing antibodies (nAbs)*, which can bind to extracellular viruses and inhibit viral entry and infection. The spike protein was therefore selected because it is an abundant surface protein of the virion and the main target for nAb against the related SARS-CoV-1 [27]. All first-generation vaccines also induce modest CD4 and CD8 T-cell responses due to the large number of T-cell epitopes within the spike protein and within other structural antigens included in vaccines based on inactive forms of the virus. Two widely employed vaccines used mRNA platforms to deliver spike (Spikevax-mRNA-1273, Moderna; and Comirnaty-BNT162b2, Pfizer-BioNTech), and three vaccines used nonreplicative adenoviral vectors (Vaxzevria/Covishield/AZD1222/ChAdOx1-spike, AstraZeneca/Serum Institute of India; Ad26.COV.2, Johnson & Johnson/Janssen; and Ad5-nCoV, Cansino Biologics; reviewed in [28]). Later additions to the global vaccine repertoire included whole inactivated viruses (CoronaVac, Sinovac; Covilo/BBIBP-CorV, Sinopharm; and Covaxin/BBV512, Bharat Biotech) and subunit proteins (Covovax, Serum Institute of India; and NVXCoV2373, Novavax), which were also based around ancestral sequences.

Widespread rollout and unprecedented research interest have offered ample opportunity for studies into the optimal *vaccine regimen* for protection. Notable findings from these studies include the qualitative differences in immune response induced by different platforms (e.g., Ad vs. mRNA vs. inactive virus) or different heterologous prime-boost regimens; the effects of vaccine doses and the timing of boosters; and the effects of sex and age on vaccine efficacy (reviewed elsewhere [5, 29, 30]). This knowledge will be informative for the design of vaccines against other infectious agents.

Vaccine efficacy studies determine how protective vaccine regimens are in a given cohort *using defined end points*, e.g., efficacy against symptomatic infection or against admission to intensive care. Protection against infection is the ultimate goal of vaccines, thus preventing the disease among individuals and breaking the chain of onward transmission. Many vaccines do not reach this high-bar, but they can still limit the severity of disease in those who become infected, thus protecting against hospitalization or death. A common way to measure vaccine efficacy is to test only symptomatic individuals during a trial rather than testing everyone weekly by PCR. This strategy could not be used to determine the efficacy of vaccines against asymptomatic infection. However, it would be easier to identify and record the number of hospitalizations, intensive care unit visits, and fatalities during vaccine trials when compared to infections, thus making it more feasible to assess the efficacy of a vaccine against severe disease. It can be difficult to generalize results across diverse populations; however, the wealth of data for SARS-CoV-2 vaccines has allowed systematic reviews and meta-analyses to integrate data from millions of vaccinations, thus resulting in some consistent rules about where vaccines have been effective [5, 29, 30].

The high rates of infection globally have enabled the rapid determination of vaccine efficacy in several countries, reassuringly showing that several approaches have been highly protective against symptomatic infection [31–33]. A recent meta-analysis of 29 trials highlighted the impressive efficacy of first-generation vaccines, showing that the combined *efficacy of a full course of vaccination against hospitalization was greater than 95%* [5]. Yang et al. found that efficacy was consistent across mRNA, DNA, viral vectors, and inactive viruses and that differences in trial design, population characteristics and other factors were stronger contributors to heterogeneity than vaccine modality. Collectively, this body of evidence will be useful to build on for next-generation vaccine development.

### Need for better SARS-CoV-2 vaccines

There are several limitations to the current licensed vaccines that we will consider below. Ongoing waves of SARS-CoV-2 infection sustain the scenario where in the absence of improved vaccines or immunotherapies, clinically vulnerable individuals will still require annual or biannual vaccinations with updated immunogens matched to circulating variants.

#### Limited ability to block infections

What has become clear is that first-generation vaccines are somewhat limited in their ability to prevent infections, with an efficacy of ~75% for preventing symptomatic infections but only ~45% for preventing asymptomatic infections [5]. This is particularly the case for the sublineages of the Omicron variant, due to its ability to evade antibodies from prior exposure to both earlier variants and vaccines [16, 34, 35]. As a result, widespread breakthrough infections occur even in populations with near-universal vaccine coverage.

#### Nonresponders

For all current vaccines, there is a significant population of individuals who are considered nonresponders (i.e., nonseroconverters), particularly those with an inability to mount effective B-cell responses. These nonseroconverters include people on B-cell-depleting therapy or people with inborn errors of immunity, as these individuals are typically more susceptible to prolonged infection and higher rates of progression to severe disease [36, 37]. Despite low fatality rates globally, high rates of infection pose a persistent challenge to shield vulnerable individuals who cannot be productively vaccinated.

#### Durability

Efficacy against symptomatic infection also waned over time for all vaccines (average decrease of 13.6% per month, 95% CI 5.5–22.3 [5]; a decrease from 83% to ~22% from 1 to 5 months post vaccination across 18 studies [30]). However, efficacy against severe disease does appear more durable, with vaccines leading to a ~90% reduction in severe disease at 5 months postvaccination [30, 33]. This waning of efficacy is also observed for booster vaccination when Omicron is the dominant circulating variant, with Menegale et al. reporting only a 30% vaccine efficacy against symptomatic infection at 9 months after a booster [38]. The challenges associated with sustained vaccine efficacy include the waning of antibody responses as well as neutralization evasion [39–41].

#### Mucosal immunity

Currently licensed SARS-CoV-2 vaccines are all delivered parenterally via intramuscular injection, which leads to some limitations for vaccine roll-out and potentially for protective efficacy (Table 1). Needle use can reduce uptake due to the fear of needles, it can lead to needle-stick injuries; furthermore, when blood hygiene guidance is not followed and needles are reused, the spread of blood-borne infections can occur [42]. Alternative methods of administering vaccines, such as oral, sublingual, intranasal, and aerosol administration, could address these limitations.

The route of administration can have a profound impact on the localization of the immunity generated [43–45]. Alternative routes of administration are being investigated in the context of coronavirus vaccines with the aim of *inducing strong mucosal immunity at the site of viral infection and replication* to improve vaccine efficacy. Vaccine-induced respiratory mucosal immunity is vital for protection against SARS-CoV-1 in animal models [46], and greater numbers of total tissue-resident T cells in the lungs have been associated with protection from severe COVID-19 [47, 48]. In particular, it may be essential to have preexisting immune memory at the sire of viral exposure in the airways to block infection.

Currently licensed vaccines are not targeted to the airways, and thus, they induce limited mucosal immunity. However, SARS-CoV-2-reactive immunity can be detected in the airways after previous infection [49] or as preexisting cross-reactive immunity [50, 51]. Next-generation mucosally targeted vaccines may be able to draw T and B cells from the blood and lymphatics into the mucosa, prime de novo responses locally, or even boost immunity generated by previous infections of the airways. It has previously been suggested that mucosal immunity tends to be short-lived and that vaccines targeted to the mucosa may be less immunogenic at these sites [43], which are limitations that need to be overcome for efficacious mucosal-targeted vaccines.

#### Cross-reactive immunity

Licensed vaccines were originally designed to target the SARS-CoV-2 Wuhan-Hu-1 strain without knowledge of how global transmission and preexisting cross-reactive infection or vaccine-induced immunity would influence viral sequence evolution. *We are now in a better position to design vaccines to induce immunity that can cross-recognize current variants and other human and animal coronaviruses*.

Both Pfizer and Moderna have developed bivalent vaccines containing ancestral SARS-CoV-2 and an Omicron variant. These bivalent vaccines can induce stronger nAb titers to Omicron than first-generation vaccines [52–54]. Previous infection with Wuhan-Hu-1 has been associated with a weaker induction of Omicron-specific nAbs [55], suggesting an imprinting effect. Each antigen exposure via infection or vaccination reshapes immune memory and its potential to protect against future homologous or heterologous variants. It will be important to determine *how previous infection and vaccination affect the immune response to next-generation vaccines*. For instance, it has been suggested that Omicron bivalent vaccines predominantly recall B cells targeting ancestral SARS-CoV-2 epitopes rather than inducing de novo Omicron responses.

Mutations in Omicron may have led to a loss of B-cell epitopes, but this does not imply that novel Omicron-specific epitopes were exposed as a result, thus stressing the need for further investigation into the extent to which Omicron-specific immunity is achievable.

## What would an ideal pan-coronavirus vaccine look like?

The impact of vaccines can be considered on an individual or population level and offer different types of protection. For instance, on a personal level, the ideal vaccine will offer immunity that is sterilizing so that infection never establishes in a host. On the population level, an ideal vaccine would limit transmission. However, vaccines that limit disease still have benefits, as they prevent infections from causing severe disease and death.

If we were to design vaccines under the assumption that producing the gold-standard pan-coronavirus vaccine is achievable, we would aim to induce immunity that recognizes the *breadth of diversity across the Coronaviridae family*. Vaccines should also be designed to overcome the limitations of the current SARS-CoV-2 vaccines named above: they should *prevent infection, viral shedding, onward transmission, and disease; they should be effective in all age groups and in pregnant women; and they should offer durable protection, potentially lifelong, after one or a limited number of doses* (Table 1). Other vaccine characteristics that impact the manufacture and roll-out of the vaccine also deserve consideration (Table 1).

### What type of immune memory do we need to induce for an effective coronavirus vaccine?

Current approaches to vaccinology largely rely on trial and error, with immunogenic vaccines progressing to increasingly large human trials to determine their efficacy. A more efficient approach would be to identify precise *correlates of protection* that can be used to inform vaccine design and to benchmark vaccines. Correlates of protection can be identified by studying immunity in protected and unprotected individuals during natural infection studies but can also be inferred from postvaccination protection. Due to the complex interaction of different factors that contribute to an effective immune response, there may be more than one correlate of protection, and the mechanisms of protection during natural infection may differ from those seen after vaccination.

The wealth of data regarding the efficacy and immunogenicity of first-generation SARS-CoV-2 vaccines can support informed conclusions about the likely immune mechanisms of protection. Yang et al. found that higher *nAb, anti-RBD (receptor binding domain) antibodies and anti-spike antibodies were associated with higher efficacy* against infection and severe disease; however, there was no clear linear correlation, as a *large proportion of the heterogeneity in efficacy was not explained by antibodies* [5]. This is consistent with many animal studies and in-depth immunological studies that show, as expected, a measurable contribution to protection via non-nAb [56–58], NK cells [59], and MAIT cells [60, 61] and in particular T cells [62–65].

Prior to the roll-out of vaccines, the expansion of SARS-CoV-2-specific T cells and an interferon response were detectable in the blood even in the week preceding PCR positivity in asymptomatic and mild cases of primary SARS-CoV-2 infection [66]. In contrast, antibodies were detectable in the blood two weeks post-PCR positivity. During breakthrough infection after vaccination, T-cell and plasmablast activation occur rapidly, while nAb and memory B-cell activation occurs ~2 weeks postinfection. This pattern suggests the important role of early recall responses of T cells and non-nAbs in protection from severe disease [63, 64]. *Therefore, T-cell responses appear to be important not only in the clearance of virus and limiting disease but also in the early control of viral replication* [67].

It remains important that correlative and mechanistic studies continue to unpick the complex immune response to coronaviruses to hone-in on which aspects of immunity are most effective against coronaviruses to inform vaccine design.

### Does pan-coronavirus immunity exist?

Despite a lack of precise correlates of protection, it is clear that a pan-coronavirus vaccine will need to induce immunity that *cross-recognizes the full range of diversity* across this viral family. *What evidence is there that such immunity exists?*

RNA viruses have some of the highest mutation rates due to their use of RNA-dependent RNA polymerase rather than host proofreading machinery [18]. Genomic sections displaying sequence conservation over time within a single viral species or evolutionary conservation between members of the coronavirus family despite high mutation rates are indicative of *essential regions* of the viral genome that are functionally constrained, such as the fusion peptide within the spike S2 region [68, 69]. Therefore, a pan-coronavirus vaccine that is able to induce immunity targeting combinations of epitopes conserved across the whole coronavirus family should also protect against future variants and emerging viral species, in which these epitopes will likely be retained owing to functional constraints.

#### Cross-reactive antibodies

The Omicron variant has exemplified the difficulty of inducing cross-protective antibodies even within a single coronavirus species. What differentiated Omicron from previous SARS-CoV-2 variants was its emergence with a large panel of amino acid substitutions in the spike S1 region, which led to a loss of recognition by the RBD binding nAb. Importantly, these mutations also led to the loss of recognition by a wide panel of monoclonal antibodies undergoing testing in the clinic [70].

Due to ubiquitous exposure, most adults are seropositive to all four seasonal coronaviruses; however, it was shown early in the pandemic that this exposure did not lead to preexisting antibodies that could cross-recognize SARS-CoV-2 in most individuals [71]. In the few individuals who did have preexisting cross-reactive antibodies, these often targeted the S2 region of spike that is more conserved across coronaviruses but that predominantly induces nonneutralizing antibodies [72, 73]. Interestingly, monoclonal antibodies that target the conserved fusion peptide within S2 and display a broad reactivity profile across all SARS-CoV-2 variants and animal coronaviruses have been identified [69, 74–76]. RBD-targeting nAbs that recognize Omicron and a range of β-coronaviruses have also been identified, thus indicating key conserved RBD sites toward which vaccines can be designed [77, 78].

Similar to the case of broadly nAb to HIV, the description of rare subset of broadly targeting antibodies is not sufficient to elucidate the mechanism by which we can elicit these antibodies in a wide range of individuals by vaccination. However, it does serve as a proof-of-concept that broadly targeting immunity exists, thus suggesting potential routes of induction, which can be experimentally tested.

A particular challenge for pan-coronavirus vaccines is the diverse use of entry receptors by different coronaviruses (e.g., *ACE2*: SARS-CoV-1, SARS-CoV-2, NL63; *DDP4*: MERS; *9-O-acetylated sialic acid*: OC43, HKU1; *aminopeptidase N receptor*: 229E). Neutralizing antibodies often rely on physically inhibiting the interaction between the virus receptor binding domain and the host entry receptor; therefore, pan-coronavirus vaccines would need to target epitopes that can inhibit binding to all coronavirus entry receptors.

Although they are understudied, a longitudinal analysis of HCoV-OC43 and 229E suggested that they constantly adapt to avoid host immunity [79]; in particular, these viruses acquire mutations in S1 that lead to a ladder-like phylogenetic tree. In another study, historic sera, which were able to neutralize 229E sequences from the time of collection, failed to neutralize strains isolated 8–17 years later; furthermore, modern sera from children were more effective at neutralizing contemporary virus, thus demonstrating the difficulty of designing a vaccine against a moving target [80].

Recent analysis shows that the antibodies induced in the small population of SARS-CoV-1-exposed individuals who were subsequently exposed to SARS-CoV-2 appear to be particularly broadly cross-reactive, suggesting that exposure to related coronavirus species can select for cross-reactive antibodies [81]. This evidence lends support to the fact that mosaic antigens and/or sequential boosting of antibodies may be needed to selectively expand cross-reactive immunity.

#### Cross-reactive T cells

T cells are inherently more cross-reactive than B cells because they recognize short peptide sequences presented on the major histocompatibility complex (MHC) rather than complex structural epitopes and because they can target conserved internal proteins rather than just surface and structural proteins that are accessible to antibodies on extracellular virus. Additionally, the interaction between T cells and MHC-peptide is highly flexible, thus enabling a single T-cell receptor sequence to recognized in the region of 1 × 10^6^ different MHC-peptide targets [82].

Most SARS-CoV-2-specific T cells have been shown to be at least panvariant reactive, with epitopes being retained in currently circulating viruses [83–85]; thus, aside from a few key exceptions, T-cell escape is rare [61, 86, 87]. Antibody escape by Omicron has led to a marked drop in vaccine efficacy against infection; however, robust protection against severe disease has been maintained. In the context of low or often undetectable neutralizing activity of antibodies, the fact that vaccine- or previous SARS-CoV-2-induced immunity can retain its ability to limit viral replication and to clear infection indicates the important protective roles of non-nAbs and T cells.

Cross-viral species recognition by coronavirus-reactive T cells has been well described, with SARS-CoV-2-reactive T cells being reported in samples taken before SARS-CoV-2 circulated in humans. *Preexisting SARS-CoV-2 cross-reactive* T cells have been widely identified and are detected in up to three-quarters of individuals using sensitive assays and in vitro expansion [43, 67, 88–91]. Importantly, preexisting T cells have been shown to not only be cross-reactive to SARS-CoV-2 sequence peptides in vitro but also respond in vivo and have been associated with early control of viral infection, limited disease and stronger responses to vaccination [62, 68, 92, 93]. Many of these preexisting T cells target conserved epitopes within human coronaviruses, suggesting that they were induced by previous seasonal HCoV infection [62, 94]; however, there are other minor sources of these cross-reactive T cells [95–98].

Analyses of sequence evolution across the coronavirus family through evolutionary timescales and throughout the pandemic can help to *identify regions that are conserved and, if immunogenic, amenable to eliciting broadly reactive immunity*, especially in the context of selection pressure from increasing levels of vaccine-induced and infection-induced immunity.

Although enriched in proteins with higher levels of total conservation, there are examples of conserved immunogenic epitopes in most, if not all, viral proteins [43, 99, 100]. The RNA-dependent RNA polymerase (NSP12) and helicase (NSP13) of the replication-transcription complex (RTC) are the most conserved proteins when considering the rate of change at each amino acid site (homologies or nucleic acid diversity) over the course of the SARS-CoV-2 pandemic or conservation in sequence across HCoVs and the coronavirus family [62]. This is expected due to their essential roles in the viral reproduction cycle. However, T-cell responses to conserved nonstructural regions – especially the RTC—are subdominant in detectable seropositive infections [62]. This may be due to the low level of expression of these proteins, which are not encoded by subgenomic RNAs or present within the virion. Therefore, they are not needed in large quantities. *Overcoming low protein abundance may offer an opportunity for vaccine-induced immunity to improve the responses seen in natural infection*.

### Could vaccine-induced T cells offer protection against infection?

An ideal pan-coronavirus vaccine will limit infection and onward transmission rather than just prevent disease upon infection. However, is there evidence that *infection-blocking immunity exists against SARS-CoV-2 and other coronaviruses?*

An intensive study of health care workers (HCWs) in London from the week of the first UK lockdown showed that despite a high rate of exposure and infection relative to the general public [101, 102], the majority of individuals remained seronegative and repeatedly tested negative on PCR. This seronegativity could reflect a lack of exposure to the virus or could indicate early control of the virus upon exposure and abortive infections [67]. Abortive infections have been identified in seronegative individuals, a status confirmed with multiple spike and nucleoprotein sero-assays as well as pseudovirus neutralization. Abortive infections were detected by the coordinated expansion of SARS-CoV-2-specific T cells as well as the upregulation of a biomarker of infection—i.e., the interferon-stimulated gene IFI27—during the first pandemic wave of infections [62].

Abortive infection is a desirable outcome, as it precludes disease development and has a low risk of onward transmission due to viral replication levels being below the PCR detection threshold; furthermore, abortive infection serves as an important setting in which to study protective immunity [67]. What was striking about the T-cell response in the HCWs with abortive infection was the *enrichment for T cells targeting the conserved nonstructural proteins of open reading frame 1ab (ORF1ab), which make up the core of the RTC* [67]. An enrichment of RTC-specific T cells *was observed at recruitment* (prior to exposure) among individuals who went on to have an abortive infection relative to those who had a detectable infection. This finding suggests the direct role of RTC-specific T cells in protecting individuals from detectable infection upon exposure; therefore, *RTC-specific T cells were identified as a correlate of protection from detectable infection* [67]. In support of the idea that cross-protective immunity exists, recent HCoV infections were found to be associated with less severe COVID-19 upon infection with ancestral SARS-CoV-2 [103], and infection with SARS-CoV-2 of any variant was also associated with a reduced incidence of subsequent HCoV infection. In both cases, *cross-protection was correlated with stronger T-cell responses*, particularly NSP12/13 targeting CD8 + T cells [104].

Preexisting cross-reactive T cells could contribute to rapid viral control due to the following characteristics: their *adapted phenotype*, as they are already differentiated to memory T cells poised to respond with rapid proliferation and immediate effector function; their *location*, as they are enriched at the site of viral exposure [49, 50]; and their *frequency*, as they are already pre-expanded to higher magnitudes than in naïve individuals. For many studies, it was not possible to sample adaptive immunity pre-exposure; therefore, a combination of de novo and preexisting T-cell responses was studied and correlated with early control [96, 105], often noting an enrichment for responses to conserved epitopes postexposure [106].

Overall, there is evidence that *T cells are important in early viral control and infection blockade*, thus providing support for the inclusion of T-cell antigens within next-generation vaccines alongside B-cell antigens.

## What can we learn from universal vaccines against other viruses?

The development of broadly protective vaccines has been attempted previously, particularly for influenza and Henipavirus. Below, we will briefly discuss what can be learned from this body of literature when designing pan-coronavirus vaccines.

Henipavirus is a genus of negative-strand RNA viruses of the Paramyxoviridae family with a broad host range, primarily small mammals. These viruses have recently emerged as zoonotic infections. Hendra virus (HeV) is a bat-borne virus that causes deadly infections in both humans and animals, predominantly horses, with the flying fox identified as a key reservoir species [107]. Nipah virus is another zoonotic Henipavirus that was identified during an outbreak in 1998 that caused ~265 infections and >100 deaths in Malaysian pig farms [108].

Support for cross-protective immunity against HeV and Nipah virus comes from human and animal serology, which show cross-neutralization [109]. A HeV vaccine that was developed and licensed for horses is the first vaccine against a containment category four human pathogen; this vaccine aims to limit bat-to-horse spread, as viral amplification in horses appears to be the main source of human infections [110]. This subunit vaccine is based on the highly conserved G protein of HeV, which may explain cross-protection against Nipah virus. Henipaviruses may reflect a relatively easy target for broadly protective vaccines due to stringent requirements for the entry receptor EphB2, limited observed viral escape, and relatively low genetic diversity [111].

Influenza, on the other hand, embodies a target that is potentially even more challenging than coronaviruses, particularly due to its segmented genome, which allows reassortment in coinfected cells. This reassortment leads to antigenic shift, which is a major determinant of pandemic influenza strains. Influenza also has a broad host range and vast genetic diversity generated by error-prone replication and antigenic shift. Of the four types of influenza, only type A has caused human pandemics and can be zoonotic, while type B causes seasonal outbreaks and only circulates in humans.

To counter this constant evolution, seasonal vaccines are redesigned annually on predictions of what will be the dominant circulating variants in the upcoming ‘flu season.’ Predictions must be made significantly ahead of time owing to the time needed to manufacture sufficient doses of vaccine, which can lead to mismatches between vaccine and circulating strains that almost universally lead to limited efficacy.

More than 800 pandemic influenza vaccines have been designed and reported, with only eight being described as universal or paninfluenza [112]. A universal influenza vaccine would have to protect against currently circulating human and zoonotic strains and emerging variants resulting from antigenic drift and shift.

Influenza is highly diverse in sequence. For influenza A, there are at least 18 hemagglutinin (HA) and 12 neuraminidase (NA) subtypes, with group 1 and group 2 species sharing only ~37% sequence homology for HA and NA proteins and only ~25% sequence homology with influenza B species (reviewed in ref. [113]). Internal proteins such as the polymerase subunits (PA, PB1, PB2), matrix and nucleoprotein can have conservation in sequence of 77–97% within influenza A but much reduced conservation with influenza B species.

Two conserved antigenic targets of interest for vaccinologists are the membrane-proximal stalk domain of HA [114] and the ectodomain of the ion channel M2 [115]. Paninfluenza A/B monoclonal nAbs, such as the HA-stem targeting CR9114, have shown protection in lethal heterosubtypic influenza challenge models. However, cross-subtype HA-stem antibody responses between H1N1 and H3N2 are limited due to differences in HA-stem glycosylation sites and low sequence conservation.

Paninfluenza conserved T-cell epitopes, such as HLA-A*02-restricted PB_1413–421,_ have been identified [116]; however, these are often not immunodominant in natural infection. A vaccine based on conserved regions of M1 and NP within a modified vaccinia Ankara (MVA) vector showed encouraging immunogenicity data; however, it was not efficacious in humans [117]. Recently, a mosaic VLP vaccine containing HA and NA antigens was shown to induce nAb and T cells that could recognize a broad range of seasonal influenza strains and could protect against lethal H1N1 and H3N2 challenge in mice [118]. A vaccine based on conserved long peptides (Flu-v, PepTcell SEEK) was tested in a human challenge study with H1N1 challenge virus and reduced mild to moderate disease [119].

One important aspect of vaccine design is the durability of the protection. As with SARS-CoV-2 vaccines, immunity and efficacy have been observed to wane after influenza vaccination, with detectable loss even over the course of a flu season [120]. Immunity induced by natural infection may be more long-lasting and can be life-long against certain strains [121]. *A better understanding of how T and B-cell responses evolve over time and the signals needed to prolong protective immunity could have an enormous impact across the spectrum of vaccines*.

What is encouraging, however, when thinking about the impact vaccination could have, is the apparent eradication of influenza A subtypes from the human population linked to cross-reactive immunity. On several occasions following pandemics, influenza A cross-reactive immunity has led to a level of protective herd immunity that has eliminated viral subtypes, including H1N1 in 1957, H2N2 in 1968, and seasonal H1N1 in 2009. Universal vaccines aim to recapitulate this type of durable cross-reactive immunity but on a broader scale and without the need for infection or symptomatic disease.

Interestingly, the immunological phenomenon of original antigenic sin, where immune responses to subsequent similar but nonidentical antigens are dominated by less effective cross-reactive immunity rather than generating de novo highly specific and effective immunity, has been described for influenza. Protection against hemagglutinin subtypes similar to the strain circulating around the time of a cohort’s first influenza exposure as children appears to be stronger than against other subtypes, suggesting the strong influence of that first exposure on the immune response to all subsequent influenza exposures [122–124]. This raises the interesting question of whether vaccination prior to the first influenza infection can be tailored to shape a more broadly protective immune response throughout life [125].

It is anticipated that the development of a universal influenza vaccine will be an iterative process informed by continued studies into vaccine and natural infection correlates of protection.

## Pan-coronavirus vaccine approaches

**Fig. 2:**
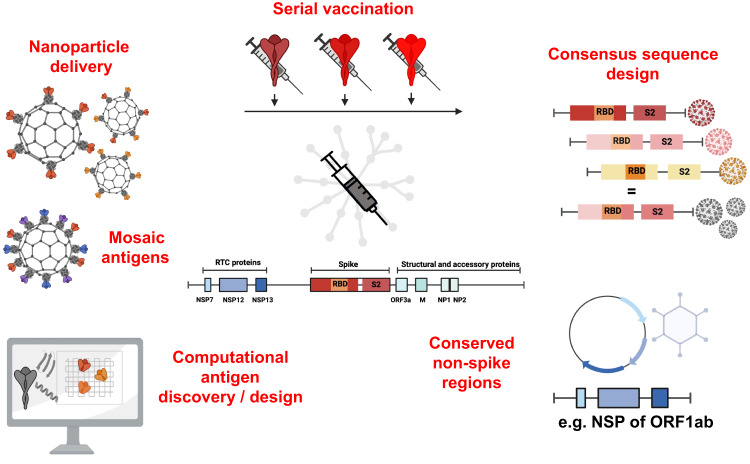
Graphic summary of next-generation approaches for broadly targeted and pan-coronavirus vaccines, including nanoparticle delivery; the use of mosaic antigens or serial vaccination to educate the immune system with multiple versions of a given viral protein; the use of novel regions of the virus, in particular conserved regions outside of spike; and finally, consensus sequence design or computational antigen design, to select the antigenic sequence that induces the most broadly reactive immunity. NP nucleoprotein, NSP non-structural protein, ORF open reading frame, RBD receptor binding domain, RTC replication-transcription complex. Created with BioRender.com

Despite decades of research and multiple approaches being pursued, there are no protective vaccines for highly variable viruses such as HIV, influenza and HCV [126]. However, advances in our understanding of anti-viral immunity, cross-reactivity, and molecular mechanisms of vaccine-induced immunity offer renewed hope. In particular, advances in computational methods have had a profound impact on the way we measure immunity and design antigens for vaccines. Below, we will highlight some of the approaches (Fig. 2**;** Table 2, *preclinical;* Table 3, *in clinical testing*) that are currently being applied to coronavirus vaccines, including many novel approaches that could lead to paradigm shifts in vaccinology.

**Table 2:** A selection of next-generation pan-variant to pan-coronavirus vaccines undergoing preclinical testing

Sponsor/funder	Breadth of target	Vaccine platform	Vaccine name	Antigen	Adjuvant	Adminstered
Duke University	β-coronavirus and Merbecovirus	Ferritin nanoparticle	RBD–scNP	RBD monovalent or trivalent	3M-052 alum	Intramuscular
Stanford University	Sarbecovirus	Ferritin nanoparticle	DCFHP	Spike (70 aa deleted)	Alhydrogel	Intramuscular
Caltech	Sarbecovirus	Mosaic nanoparticle	Mosaic-8	1 SARS-CoV-2 RBD + 7 sarbecovirus RBD	AddaVax	Intramuscular and Intraperitoneal
Caltech	Sarbecovirus	Quartet or Dual Quartet Nanocage	–	1 SARS-CoV-2 RBD + 7 sarbecovirus RBD	AddaVax	–
SK Bioscience, Uni of Washington, Uni of North Carolina at Chapel Hill	Sarbecovirus	Mosaic nanoparticle	RBD-NP or GBP511	RBD from: SARS-CoV-2 + SARS-CoV-1 + 2 serbecovirus	AS03	Intramuscular
Oragenics, Inspirevax, National Research Council of Canada	Pan-coronavirus	Protein subunit	NT-CoV2-1	SmT1v3 (version of SARS-CoV-2 spike)	BDX301	Intranasal
Francis Crick Institute	Pan-coronavirus	Protein subunit with DNA boost	–	S2	Monophosphoryl lipid A for protein	Intraperitoneal protein, Intramuscular and Intravenous DNA
INSERM Vaccine Research Institute	Sarbecovirus	Protein subunit	CD40.CoV2	RBD or Spike/NP conserved epitopes?	Anti-CD40 targeting	Intraperitoneal and Intramuscular
Valo Therapeutics Ltd	Pan-coronavirus	Adenovirus-based + HLA-matched peptides	PeptiVax	Spike + peptide (CD8 epitopes)	–	–
OSIVAX	SARS-CoV1, SARS-CoV2	Virus-like particle oligoDOM	OVX033	NP (Wuhan)	Unadjuvanted or squalene-in-water emulsion	Intramuscular
Fudan University China	Sarbecovirus	Protein subunit - human IgG Fc-conjugated RBD	CF501	RBD-Fc	CF501 (sting agonist)	Intramuscular
University of California, Irvine	Pan-coronavirus	Viral vector AAV-9	–	Multi-epitope	CXCL11 'prime-pull'	Subcutaneous and Intranasal
University of North Carolina at Chapel Hill	Sarbecovirus	Alphavirus	VRP3526-spike	Spike	–	Footpad
University of Wisconsin/Madison	Pan-coronavirus	VLP nanoparticle	VLP-S2	S2	Various (AddaVax, Qs-21, AddaS03, pIC)	Subcutaneous
Universitätsmedizin Berlin	Pan-coronavirus	Non-integrating lentivirus	NILV-PanCoVac	T cell epitopes in structural proteins	–	Intranasal

**Table 3:** Highlights of next-generation pan-variant and pan-sarbecovirus vaccines undergoing early clinical testing

Sponsor/funder	Breadth of target	Trial number	Phase	Vaccine platform	Vaccine name	Antigen	Adjuvant	Adminstered
WRAIR	Sarbecovirus	NCT04784767	I	Ferritin nanoparticle	SpFN_1B-06-PL	Spike	ALFQ	Intramuscular
DIOSynVax, PharmaJet, University of Southampton	Sarbecovirus	ISRCTN87813400	I	mRNA	pEVAC-PS	T2_17 (DIOSynVax generated)	–	Intradermal needle-free
VBI Vaccines Inc.	SARS-CoV-2, SARS-CoV, and MERS-CoV	NCT05548439	I	Enveloped virus-like particle (eVLP)	VBI-2901	Spike trivalent (SARS-CoV-1, SARS-CoV-2, MERS-CoV	Aluminum phosphate (Alum)	Intramuscular
Gristone Bio	Sarbecovirus/Pan-variant	NCT05148962	I	Self-amplifying mRNA	GRT-R910	Codon optimized, prefusion stabilized Spike + T cell epitopes	–	Intramuscular
NIH	Pan-variant	NCT06026514	I	Live-attenuated bovine/human parainfluenza	B/HPIV3/S-2P	HexaPro Spike	–	Nasal spray
Tetherex & Mayo clinic	Pan-variant	NCT04839042	I	Single cycle Ad	SC-Ad6-1	Spike trivalent cocktail	–	Intranasal and Inhaled

### Homotypic nanoparticle delivery

Nanoparticles are nanoscale particulate structures that mimic the structural features of natural viruses (Fig. 2). In recent years, the development of nanoparticle-based vaccines has benefitted from advances in material science that have led to improvements in antigen structure and stability, targeted vaccine delivery, and immunogenicity with good safety profiles. Nanoparticles can be used to encapsulate the viral antigen into the nanoparticle core (e.g., mRNA lipid nanoparticles), or they can present the antigen as protein on the surface of the nanoparticles (e.g., virus-like particles, protein nanoparticles).

For example, nanoparticles have been constructed with individual or multiple versions of the full spike protein or RBD region and presented in high-order antigen arrays in native-like conformations (reviewed in ref. [127]). Fusing naturally occurring bacterial ferritin to antigens as a vaccine delivery platform increases the immune response for weakly immunogenic targets and recapitulates the complex structure of trimeric class I glycoproteins. This makes *ferritin nanoparticles* a promising avenue to explore to generate enhanced immune responses. A group at the *Walter Reed Army Institute of Research* *(WRAIR)* created prefusion-stabilized *S-trimer-ferritin nanoparticles (SpFNs)*, which were shown to be stable candidate immunogens that elicited potent nAb titers against SARS-CoV-2 variants and robust protection against challenge in a K18-hACE2 mouse model [128, 129] (Table 3). Heterologous prime-boost vaccine regimens combining SpFN with Johnson & Johnson’s adenoviral vectored vaccine Ad26.COV2.S induced strong nAb and T-cell responses that protected mice from heterologous Omicron BA.5 challenge [130].

The immunogenicity of the RBD may be enhanced by presenting multiple copies of the RBD on a single particle to B cells, allowing multiple antigen-BCR engagements. A group based at *Duke University* took a similar approach; they used self-assembling *Helicobacter pylori* ferritin nanoparticles with 24 copies of RBD attached via sortase A, named *RBD–scNP* [131] (Table 2). Using the TLR7/8 agonist 3M-052 alum as an adjuvant, RBD-scNP was designed to be a pan-β-coronavirus/merbecovirus vaccine. RBD-scNPs provided sterilizing immunity in the upper airways of macaques and induced nAbs that could cross-recognize bat WIV-1, bat SHC014, SARS-CoV-1, and pangolin COV GXP4L viruses [131] as well as all SARS-CoV-2 variants tested [132]. An updated trivalent version encoding the RBD from SARS-CoV-2, bat RSHC014, and MERS-CoV induced nAb to MERS-CoV and SARS-CoV-2 Omicron [133]. This vaccine is being manufactured at the Duke Human Vaccine Institute for Phase-I testing in humans.

Another ferritin nanoparticle-based vaccine is *Delta-C70-Ferritin-HexaPro (DCFHP)* from *Stanford University* *(*Table 2). It is based on SΔC-Fer, which displays a truncated form of the prefusion SARS-CoV-2 Wuhan-Hu-1 spike ectodomain trimer on self-assembling nanoparticles. Due to its use of a stabilized spike protein (HexaPro, discussed below), DCFHP is stable at temperatures ranging from 4 to 37 °C for at least 14 days, making local distribution of this vaccine feasible without refrigeration [134]. It also contains a deletion of 70 amino acid residues from the C-terminus, a region that is highly flexible and that contains an immunodominant linear epitope that is strongly targeted by non-nAbs in convalescent COVID-19 plasma. The removal of this region and the multivalent presentation on a ferritin nanoparticle significantly improved DCFHP’s neutralizing potency in mice relative to the use of unedited spike, since it allows the immune system to ‘not get distracted’ by creating an antibody response against a region that is ultimately nonneutralizing [134]. Wuhan-Hu-1 was specifically chosen to be expressed in the vaccine after the group reported that strain-specific mutations adversely altered the antigen structure, stability and immunogenicity [135]. In both mice and rhesus macaques, a two-dose intramuscular immunization regimen resulted in durable, robust, and broad neutralization against all SARS-CoV-2 variants tested, including the Omicron subvariants BA.4/539 and BQ.140. Antisera from immunized macaques also showed durable neutralization against SARS-CoV-1.

A collaboration between *Oragenics, Inspirevax, and the National Research Council of Canada* has led to the development of *NT-CoV-2* (Table 2). To mimic the trimerized conformation of the native SARS-CoV-2 spike glycoprotein more closely, they fused naturally trimerized human resistin to the spike ectodomain to generate a stable trimer protein antigen called SmT1. Immunized serum from mice vaccinated with SmT1 strongly blocked the binding of SARS-CoV-2 alpha and beta variants to cells [136]. To engage mucosal immunity, a modified version of SmT1 was combined with the mucosal adjuvant BDX301 (named *NT-CoV2-1*). BALB/c mice immunized with NT-CoV2-1 generated strong IgA and IgG responses in both BAL and serum, as well as strong neutralization responses against SARS-CoV-2 variants [137].

The French vaccine company *OSIVAX* has employed self-assembling nanoparticles to deliver the nucleocapsid protein (NP) rather than spike/RBD for the induction of T cells and binding antibodies, both of which have been associated with protection from severe disease in humans [59, 138] (Table 2). Using the ancestral Wuhan-Hu-1 sequence NP in their vaccine *OVX033*, they showed cross-protection in mice against Delta and Omicron BA.1 strains [139]. Vaccines using whole NPs have also been attempted with the Ad5 vector [140].

### Mosaic antigen delivery

Combining several RBD regions for presentation as a vaccine, especially from both humans and animal coronaviruses, increases the likelihood of eliciting cross-reactive antibody responses. A group from *California Institute of Technology (Caltech)* has utilized a multivalent engineered protein domain called SpyCatcher to fuse antigens to virus-like particles. SpyCatcher003-mi3 nanoparticles that display RBDs from both human and animal coronaviruses were prepared to evaluate the effectiveness of mosaic particles in producing cross-reactive antibody responses (Table 2). RBDs from Bat CoV RaTG13, Bat CoV SHC014, Bat CoV Rs4081, Bat CoV RmYN02, Bat CoV Rf1, Bat CoV WIV1, Pangolin CoV Pang17, and SARS-CoV-2 were expressed on SpyTag proteins, which were then fused to one SpyCatcher protein with multiple domains, leading to the generation of the *mosaic-8b* nanoparticle vaccine [141].

Although there was no significant difference in the magnitude of the *total* anti–SARS-CoV-2 IgG responses between homotypic and mosaic immunizations, more *cross-reactive* antibody responses were observed for mosaic-8b immunizations [142]. For instance, mosaic-8b vaccination induced nAb responses to SARS-CoV-1 despite its RBD not being included in the vaccine [141]. Moreover, mosaic-8b protected against SARS-CoV-2 Delta as well as SARS-CoV-1 challenge in nonhuman primates [142]. Monoclonal antibodies (mAbs) capable of neutralizing human and animal sarbecoviruses and SARS-CoV-2 variants could be isolated from mosaic-8b-vaccinated mice [143].

Due to challenges in scaling the production of 9 different vaccine components, the group is currently developing *quartet nanocages* encoding RBDs from SHC014, Rs4081, RaTG13 and SARS-CoV-2 Wuhan-Hu-1 (Table 2). Instead of one RBD type occupying a domain, each domain is occupied by all four RBD types in a quartet nanocage. Quartet nanocage immunizations were able to induce antibody responses in mice to diverse sarbecoviruses, although with less breadth than mosaic-8b vaccination [144]. The development and testing of nanocages is ongoing to ensure readiness for clinical trials.

*Washington University and SK Biosciences* took a similar approach, genetically fusing SARS-CoV-2 RBD on the exterior surface of the two-component protein nanoparticle I53-50, which is a computationally designed 120-subunit complex with icosahedral symmetry constructed from trimeric (I53-50A) and pentameric (I53-50B) components. RBDs from SARS-CoV-2, SARS-CoV-1, bat CoV RaTG13, and bat CoV WIV1 were genetically fused using linkers to I53-50A, allowing the display of 60 RBDs in a trimeric form in a vaccine named *RBD-NP or GBP511* [145] (Table 2). Serum from RBD-NP-immunized nonhuman primates strongly cross-reacted with Pangolin-GD and RaTG13 RBDs but had weaker binding to distantly related RmYN02, SARS-CoV-1, WIV16, and ZXC21 RBDs [146]. Fifteen broadly neutralizing mAbs were isolated from macaques that could neutralize a panel of pseudoviruses carrying spike proteins of SARS-CoV-2 variants, including Omicron BA.1 and BA.4/5, and SARS-CoV-1 [147].

*VBI Vaccines* is a biopharmaceutical company developing vaccines that mimic the natural presentation of the virus to elicit innate immune responses, such as with their proprietary enveloped virus-like particle (eVLP) technology (Table 3). They used this technology to design *VBI-2901*, a vaccine that expresses a modified prefusion form of spike proteins from SARS-CoV-2, SARS-CoV-1, and MERS-CoV. Mice vaccinated with VBI-2901 elicited a strong neutralization response against all variants and Bat RaTG13. Compared to its sister vaccine VBI-2902, which only contains the ancestral Wuhan-Hu-1 spike, VBI-2901 generated a 2.5-fold stronger response to the ancestral strain and a ninefold stronger response against the bat coronavirus [148]. VBI-2901 is being examined in Phase-I trials.

Interestingly, some mosaic vaccines employ organized repeated patterns of antigens, such as RBD-NP/GBP511, while others use randomly distributed antigens, such as mosaic-8b. In theory, random distribution should preferentially engage cross-reactive B cells that can cross-recognize multiple versions of RBD/spike proteins next to each other on the nanoparticle. However, it remains unclear which approach is most effective.

### Serial vaccination

Rather than trying to present multiple antigens together in the form of a mosaic vaccine to the immune system, an alternative approach could be to sequentially vaccinate with different antigens in a series in an attempt to boost the most cross-reactive immunity (Fig. 2). Groups at *Yale University* tested this approach with their lipid nanoparticle *(LNP)-mRNA* vaccines encoding the full-length spike of SARS-CoV-2 Delta, SARS-CoV-1, and MERS-CoV. A direct comparison in mice demonstrated that sequential prime-boost vaccination with their three constructs (6 vaccinations in total) induced a stronger antibody response than simultaneous vaccination (prime and then boost with a mixture of all three, 2 vaccinations in total) [149]. It is not surprising that separating the vaccines over time leads to a stronger response; however, there was also some indication that it induced greater cross-reactivity and durability of the immune response [149]. It would be interesting to see if a longer time interval between vaccinations than the 3 weeks employed in this study changes the magnitude and type of immune response generated, as it does when reboosting with Ad/MVA encoding HCV antigens [150].

### Consensus sequence

One approach that has been taken is to design vaccine antigens that are enriched for conserved T and B-cell epitopes either as a consensus sequence (which is the closest natural circulating sequence to a bioinformatically calculated consensus) or as a string of epitopes taken out of their natural context within the viral protein (Fig. 2).

A number of epitopes from structural and nonstructural viral proteins conserved across a curated subset of β-coronaviruses (human and animal [100]) have been combined into an antigen for delivery in an adeno-associated viral vector that has shown pan-SARS-CoV-2 variant protection in mice [151]. Interestingly, T-cell responses to the 22 conserved epitopes within this vaccine were enriched in individuals with asymptomatic infections relative to individuals with symptomatic infection [151].

### Computational identification of cross-reactive immunogens

An important and exciting emerging field in vaccinology is the use of computationally designed vaccine antigens rather than a circulating viral sequence (Fig. 2). Several different approaches are being tested to bioinformatically design novel antigen sequences using large amounts of viral sequencing data integrated with other sources of information, such as mapped epitopes and protein structures. These antigens aim to outperform any sequences selected from a circulating virus in their induction of broadly protective immunity, often by targeting the immune response to conserved but subdominant epitopes.

The most clinically advanced approach is that taken by *DIOSynVax* (Digitally Immune Optimised Synthetic Vaccines), who have partnered with PharmaJet for a first-in-human needle-free intradermal vaccine *pEVAC-PS* encoding their synthetic antigen T2_17 based on coronavirus RBD sequences (Table 3) [152]. DIOSynVax’s approach is a viral-genome-informed method that creates an antigen sequence that is as similar as possible to all inputted sequences from a phylogenetic perspective—in this case, representative sequences for all known sarbecoviruses. This sequence is modified to ensure that key antibody epitopes are retained. Their antigen has been tested using several different platforms, including DNA, MVA and mRNA. Despite being designed before the emergence of several variants, including Delta and Omicron, the antigen induces cross-reactive nAb to these and several tested sarbecoviruses in mice, rabbits, and guinea pigs [152].

Relatedly, Hie et al. used machine learning language models to “learn” the language of viral escape. They have applied their constrained semantic change search framework to the analysis of escape to influenza A HA, HIV envelope, and SARS-CoV-2 spike to learn structural patterns of antibody escape [152]. This could lead to the prediction of future escape variants and the design of vaccine antigens that encode structural epitopes that are more difficult to escape.

An alternative approach to identifying conserved regions by comparing linear viral sequences is to use structural information on how regions interact in the 3D structure of the viral protein. This can identify “networked” regions that are so essential to a protein’s structure that they cannot be mutated without loss of structure and functionality [153]. Nathan et al. used this approach to identify CD8 epitopes within mutation-resistant networked regions that were commonly targeted in convalescent individuals [153].

### Combining T-cell and antibody antigens in one vaccine

Although many of the vaccine approaches discussed above will induce both T and B cells, it is often difficult to fully optimize the vaccine platform and immunogen itself for both T and B-cell engagement. However, several approaches are being tested, including the combination of separate antigens to elicit both T and B-cell responses. For instance, once they are identified, short sequences corresponding to broadly conserved CD4 and CD8 epitopes can often be added to vaccines encoding carefully designed structural antigens that are used to elicit B-cell responses. Charged nonreplicative adenoviruses that encode the full-length spike for antibody induction (analogous to AZD1222) have been coated with peptides corresponding to conserved CD8 T-cell epitopes [154].

*Gritstone Biotech* employed Venezuelan equine encephalitis virus-based self-amplifying mRNA vector in a lipid nanoparticle called *GRT-R910* encoding both the prefusion stabilized full-length SARS-CoV-2 spike and additional conserved T-cell epitopes from ORF3a, NP and membrane (Table 3). Self-amplifying mRNA generates high and more durable expression of the encoded coronavirus antigens, which could translate to greater immunogenicity than nonamplifying mRNA vaccines [155, 156]. GRT-R910 protected against SARS-CoV-2 infection in macaques when used as a homologous prime-boost or as a single boost to a chimpanzee adenovirus-encoded spike [157]. Encouraging results have been published for Phase-I in healthy adults aged >60, showing nAb responses to SARS-CoV-2 Beta, Delta, and Omicron BA.1 at 6 months after GRT-R910 vaccination administered as a booster 19-31 weeks after a primary vaccination series with AZD1222 (ChAdOx1-spike) [158]. GRT-R910 also expanded the breadth and magnitude of the T-cell response to all antigens included. A 10,000-participant Phase-IIb trial will be conducted as part of the COVID-19 Prevention Network.

### Other noteworthy approaches

In addition to adjusting the immunogen sequence itself, using more immunogenic vaccine delivery platforms and adjuvants could enhance the total coronavirus-specific immune responses generated, boosting broadly targeted responses. For instance, an IgG-Fc conjugated ancestral Wuhan-Hu-1 RBD sequence adjuvanted with a novel STING agonist CF501 induced potent nAb activity against SARS-CoV-2 Omicron and XBB variants in macaques, mice, and rabbits [159, 160] (Table 2).

Similarly, during a malaria vaccine research program in 1986, WRAIR developed the adjuvant ALFQ, which consists of liposomes containing saturated phospholipids, cholesterol, monophosphoryl A and saponin QS21, which have been proven to be potent and safe [161]. ALFQ-adjuvanted SpFNs elicit superior binding and neutralization responses and a stronger, more polyfunctional Th1 response than the adjuvant Alhydrogel in mice and macaques [128, 162, 163].

A rather simple but very effective method of removing dominant epitopes to enhance responses to conserved subdominant epitopes involved the removal of the S1 portion of spike containing the RBD and then the administration of a vaccine with only S2 as a DNA vaccine or protein [73] (Table 2). The responses to S2 were stronger than when full-length spike was used as the antigen; furthermore, the S2-only vaccine was shown to induce nAb to both α- and β- coronaviruses (presumably to the fusion peptide as has been described in humans [69]), and it boosted a more broadly targeting antibody response overall than repeated homologous full-length spike vaccines [73]. Moreover, nonneutralizing binding antibodies to S2 have been linked to vaccine-mediated protection in a murine model using an alphavirus vectored pan-sarbecovirus [72]. Virus-like particles encoding S2 have also been tested and could induce nAbs to all human coronaviruses in Syrian hamsters (HCoV, SARS-CoV-1 and SARS-CoV-2 variants) (Table 2). VLP-S2 reduced viral replication in respiratory tissues in hamsters challenged with SARS-CoV-2 Beta, Delta, and Omicron BA.1 variants and against a pangolin coronavirus [164].

Structure-guided spike design has also been used to identify artificial substitutions in the spike sequence that can *increase the expression, solubility, and stability* of spike (termed HexaPro) for use as a vaccine antigen [165, 166]. This HexaPro antigen was used in a regimen termed “prime and spike”, in which a priming dose was administered intramuscularly to induce peripheral immune memory and a boosting dose was administered intranasally to expand and recruit spike-specific T and B cells to the mucosa [167]. This approach laid down tissue-resident immunity within the respiratory mucosa, with the hope of offering more rapid and efficacious recall responses on exposure to sarbecoviruses. Intranasal delivery using a lung-tropic adeno-associated virus 9 vector to prime and chemokine pull (CXCL11) effectively induced SARS-CoV-2-specific lung-resident T cells and offered better protection against SARS-CoV-2 in mice [168] (Table 2). Alternative technologies for administering vaccines may yield greater mucosal targeting of immunity. For example, a live-attenuated parainfluenza vectored spike vaccine (B/HPIV3/S-6P) will be tested using a nasal spray for mucosal targeting after promising results were observed in nonhuman primates and hamsters [169, 170] (Table 3). The research company ISR has developed an inhaled dry powder vaccine that does not require needles of cold chains [171], and researchers at Yonsei University have developed a sublingual dissolving microneedle [172]. Both of these vaccines use ancestral SARS-CoV-2 S1 antigens (Table 2).

The American pharmaceutical company *Tetherex and the Mayo Clinic* developed a replicating adenoviral vector (*SC-Ad6-1*) that encodes a full-length spike; this vaccine is administered intranasally, and it aims to induce mucosal immunity [173] (Table 3). The single-cycle adenovirus replicates its genome (including the encoded spike transgene) 10,000 times but does not produce infectious virions. SC-Ad6-1 was shown to produce 100-fold more spike protein and provided better protection in hamsters than a replication-defective Ad [173]. SC-Ad6-1 has entered Phase-I testing in Australia.

Few of the vaccines described above have been tested in challenge models against viruses other than SARS-CoV-2 or have had the breadth of cross-reactive immunity they elicit rigorously tested against the full spectrum of coronaviruses. However, with multiple novel and exciting approaches to antigen design and delivery in testing, we are likely to obtain considerable knowledge regarding cross-protective immunity and the feasibility of pan-coronavirus vaccines.

## Conclusions and perspectives

A major challenge of developing vaccines against variable viruses, such as coronaviruses, is the difficulty in inducing immunity that cross-recognizes and protects against the full spectrum of possible viral variants while also limiting the opportunity for immune escape. As a result, considerable attention is being devoted to both the type of antigens included in a vaccine, as well as the sequence of antigenic targets. In particular, computational approaches, including bioinformatics and machine learning, are being used to better define sequence diversity/conservation across viruses and to aid in antigen design.

Whereas first-generation vaccines rely mainly on spike, there is strong evidence that adaptive immunity to nonspike antigens contributes to protection from infection and disease in natural infection. This comprehensive immune response should be exploited in next-generation vaccines to complement antibodies in the global population, which is currently overwhelmingly seropositive against SARS-CoV-2 spike following vaccination and/or infection. In particular, T-cell antigens outside of spikes may be important for achieving early targeting of viral replication and broad reactivity [62, 105]. Furthermore, how to combine these different antigens or epitope strings into a single vaccine is not a trivial question. There is much to be learned about how antigen processing and flanking sequences influence antigen presentation and how responses to different epitopes interact to determine immunodominance.

Further research is needed to elucidate the basic mechanism underpinning T and B-cell recruitment into an immune response, to examine antibody affinity maturation and to identify the determinants of the immunodominance hierarchy for T and B-cell epitopes, which ultimately may enable vaccine antigens to be rationally designed to induce broadly targeted immunity. A greater understanding of the correlates of protection against infection and disease is needed, particularly for T-cell responses, which are understudied due to the requirement for large sample volumes and the use of low-throughput assays. It is difficult to design a vaccine that can induce both strong antibody and T-cell responses, but it is likely that a coordinated effort employing both arms of the adaptive immune response will be most effective. Combinations of immunogenic vaccines, for instance, in heterologous prime-boost regimens, multiplexed vaccines, or serial vaccination, may be the key to achieving robust and durable immunity.

It remains unclear whether true pan-coronavirus immunity exists. Research is ongoing to determine whether individual or combined T or B-cell responses can recognize the breadth of human coronaviruses. Testing vaccine approaches and challenging the immune system with a range of viruses is the only way to determine how cross-protective immunity can be. Different research groups and funders have different priorities, ranging from more immediate protection against all variants of single species, such as SARS-CoV-2, to the long-term ambitious goal of protection against future pandemics. Fortunately, we have a panel of novel tools and approaches that can be tested against these different goals in their respective time scales.

The list of emergent viruses of concern issued by the WHO contains “disease X”, which underlines the importance of developing modular antiviral and vaccine technologies where minimal reprogramming is needed to redirect the platform to a newly emerging pathogen. One approach could be to design a suite of candidate pan-family vaccines, such as pan-coronavirus vaccines, that can be rolled out rapidly in the event of a new viral emergence to identify the candidate that best covers the novel human virus.

The durability of vaccine protection is another key area that requires a better understanding of the fundamentals of immune memory, a key question being “*how can we limit the acute contraction of the adaptive immune response after prophylactic vaccination and limit attrition of long-term memory*, in particular for nAb titers in the blood?”.

Another difficulty associated with vaccination against viruses targeting the upper respiratory tract is the generation of long-lasting mucosal immunity. Mechanistic studies in animal models suggest that mucosal targeting is likely to offer better protection against respiratory infections [43], but methods for inducing mucosal immunity are in the earliest stages of testing. The route of vaccine administration could be critical, with intranasal, aerosolized, subinguinal and oral approaches being tested; however, immunogenicity may be hampered owing to the natural immune barriers present at these sites.

Better animal models are needed in order to recapitulate human disease, diverse antigenic history (mixed infection and vaccination) and immune phenomena such as original antigenic sin and imprinting. Due to unpredictable rates of infection and diverse antigenic history, it is challenging to identify cohorts in which vaccine efficacy can be determined. The use of controlled human infections is an exciting area that has yielded new insights into the natural immune response to coronaviruses [174, 175].

Now is the right time to develop a pan-coronavirus vaccine due to advances in vaccine technologies, scalability, and immunogenicity, as well as our greater understanding of protective and cross-reactive immunity. Progress will be iterative, but with each new vaccine design, we are learning more about the feasibility of universal vaccines and how best to achieve this goal.
